# Immobilization of HRP in Mesoporous Silica and Its Application for the Construction of Polyaniline Modified Hydrogen Peroxide Biosensor

**DOI:** 10.3390/s90604635

**Published:** 2009-06-12

**Authors:** Chien-Chung Chen, Jing-Shan Do, Yesong Gu

**Affiliations:** Department of Chemical and Materials Engineering, Tunghai University, Taichung 40704, Taiwan; E-Mails: g913125@thu.edu.tw (C.C); jsdo@thu.edu.tw (J.D.)

**Keywords:** polyaniline, SBA-15, horseradish peroxidase, hydrogen peroxide, biosensor

## Abstract

Polyaniline (PANI), an attractive conductive polymer, has been successfully applied in fabricating various types of enzyme-based biosensors. In this study, we have employed mesoporous silica SBA-15 to stably entrap horseradish peroxidase (HRP), and then deposited the loaded SBA-15 on the PANI modified platinum electrode to construct a GA/SBA-15(HRP)/PANI/Pt biosensor. The mesoporous structures and morphologies of SBA-15 with or without HRP were characterized. Enzymatic protein assays were employed to evaluate HRP immobilization efficiency. Our results demonstrated that the constructed biosensor displayed a fine linear correlation between cathodic response and H_2_O_2_ concentration in the range of 0.02 to 18.5 mM, with enhanced sensitivity. In particular, the current approach provided the PANI modified biosensor with improved stability for multiple measurements.

## 1. Introduction

Polyaniline (PANI) has been used in the fabrication of various types of enzyme-based biosensors because of its porous structure, as well as its adequate conductivity and thermal stability [1-6]. To stabilize the immobilized enzyme in the matrix of PANI film, glutaraldehyde (GA) is usually employed as a bifunctional agent to crosslink enzyme molecules [7-10], but the crosslinking efficiency under standard conditions is not always satisfactory [10,11], which results in the lower sensitivity and poor stability of the resulting biosensor. Previously, we have electrochemically synthesized the PANI film on a Pt electrode in the presence of bovine serum albumin (BSA), a lysine-rich enzyme, which provides extra free *ε*-amino groups for the further crosslinking of HRP with glutaraldehyde and has significantly improved the effectiveness of a PANI modified biosensor [1]. Nevertheless, to enhance the efficiency and stability of enzyme immobilization on an electrode is still the major issue of fabricating enzyme-based biosensors.

Over the past few years, immobilizations of enzymes in well-defined mesoporous silica materials have been proven to be promising for enhancing the thermal stabilities and maintaining the catalytic activities of enzymes [12-14]. Enzymes entrapped inside the silica mesopores are less susceptible to pH and temperature alternations and organic solvents as well [12,15,16]. Among them, SBA-15, which is synthesized in the presence of nonionic triblock copolymer P123 as a template under acidic conditions, exhibits well-ordered hexagonal pore arrays of uniform pore size [17,18]. Meanwhile, SBA-15 possesses a large surface area and internal silanol hydroxyls that have affinities for physical adsorption of enzyme molecules [19-23]. Recently, SBA-15 has been successfully employed to entrap glucose oxidase (GOD) to construct a glucose biosensor, achieving with enhanced sensitivity, long-term stability and reproducibility [24,25]. In addition, monoclonal antibodies were immobilized in SBA-15 for the detection of antigen (cTnI) in the serum of patients, which is more convenient and superior to the conventional enzyme-linked immunoadsorbent assay (ELISA) [26]. For the detection of hydrogen peroxide (H_2_O_2_), SBA-15 loaded with hemoglobin (Hb) has shown a fast amperometric response, a low detection limit, and good stability [27].

In this study, we further exploited the application of mesoporous SBA-15 in entrapping HRP and constructed an amperometric GA/SBA-15(HRP)/PANI/Pt biosensor by immobilizing the SBA-15(HRP) on the electrochemically synthesized PANI film on a Pt electrode. The composite biosensor was then characterized and evaluated for the detection of H_2_O_2_ with cyclic voltammetry. In addition, its linear correlation, sensitivity and stability were investigated.

## 2. Results and Discussion

### 2.1. Characterizations of SBA-15 Mesoporous Silica

**Figure 1.**
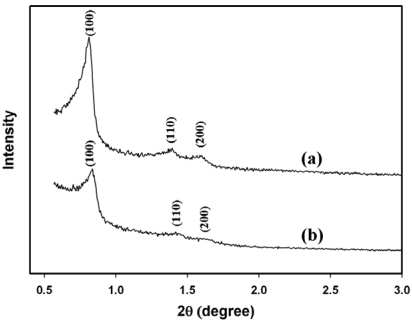
XRD pattern of (a) SBA-15 and (b) SBA-15(HRP).

**Figure 2.**
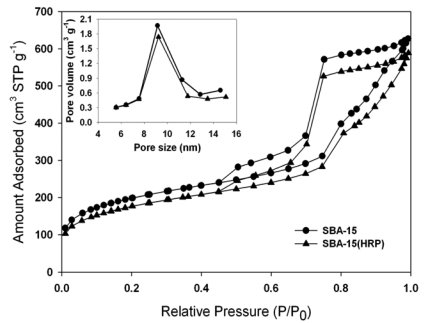
Nitrogen adsorption and desorption isotherms of SBA-15 before (●) and after (▲) the immobilization of HRP. Inset indicated the pore size distribution of SBA-15 (●) and SBA-15(HRP) (▲).

The X-ray diffraction (XRD) pattern of calcined SBA-15 [line (a) in Figure 1] revealed well-resolved peaks of 2θ at 0.812, 1.392 and 1.588, which represented the characteristic (100), (110), and (200) reflections of hexagonal mesoporous materials with *p6mm* symmetry [17]. The total specific surface area, total pore volume, and BJH pore diameter of the SBA-15 were estimated by N_2_ adsorption-desorption isotherm (Figure 2) and Barrett-Joyner-Halenda (BJH) calculation (Table 1).

**Figure 3.**
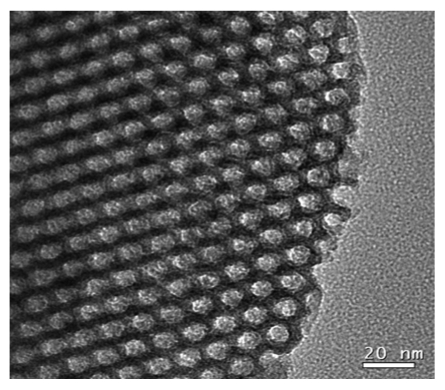
The TEM image of SBA-15.

Figure 3 shows the TEM image of calcined SBA-15 with well-ordered hexagonal array mesopores. The pore diameter was approximately 100 Å, which was close to the center of the pore size distribution (*ca.* 92 Å) shown in the inset of Figure 2. The pore size distribution indicated the major pore diameter of mesopores ranged between 80 and 110 Å, which permitted the easier access of HRP molecules because the dimension of the native HRP (MW: ∼ 44 kDa) in a neutral buffer solution was predicted to be 62 × 43 × 12 (Å)^3^ by a scanning tunneling microscopy (STE) study [28]. Meanwhile, the average pore diameter of SBA-15 estimated by the BJH method was *ca.* 76 Å (Table 1), which was below the major pore size distribution (80∼110 Å), suggesting the presence of micro-channels in the interior of SBA-15.

### 2.2. Immobilization of HRP in SBA-15 Mesopores

When compared with SBA-15, the total specific surface area and the total pore volume of SBA-15(HRP), decreased modestly by about 11% and 8%, respectively, indicating the successful entrapment of HRP within the pores of SBA-15 (Figure 2 and Table 1). The loading of HRP in SBA-15 was also confirmed with ABTS enzymatic assay, which nearly 395 units of HRP was stably retained by one gram of SBA-15 with the procedure described in the Experimental section. On the other hand, the XRD pattern of SBA-15(HRP) [line (b) in Figure 1] matched with that of unloaded SBA-15 [line (a) in Figure 1] although the intensity was decreased, and exhibited the similar mesoporous parameters listed in Table 1, suggesting SBA-15(HRP) had an analogous mesoporous structure to that of SBA-15. Furthermore, the similar pore distribution of SBA-15 and SBA-15(HRP), shown in the inset of Figure 2, implied that the retained HRP did not block the entrances of mesopores, but rather resided in the inner space of mesopores. This conclusion was further supported by the identical BJH pore diameters before and after loading of HRP (Table 1).

Furthermore, we utilized the Coomasie Brilliant Blue R-250, which is commonly employed to stain proteins in SDS-PAGE gel analysis, to stain SBA-15 and SBA-15(HRP), respectively. Both were then washed with de-staining solution several times. Our result showed that the blue dye was retained by SBA-15(HRP), but not by SBA-15, suggesting HRP was stably immobilized in SBA-15(HRP). In order to investigate whether the immobilized HRP in SBA-15(HRP) was entrapped inside the pore of SBA-15 or was adsorbed on the surface of SBA-15, we examined another mesoporous silica, MCM-41, which was synthesized by a similar approach as that used for SBA-15, but its pore diameter was estimated as 25 Å, therefore, HRP was supposed to be completely excluded by MCM-41. We found that the adsorption of HRP on the surface of silica MCM-41 was relatively unstable and most of the blue stain was washed away. Therefore, the stably immobilized HRP in SBA-15(HRP) was most likely entrapped inside the pores of SBA-15.

### 2.3. The Surface Morphology of Electrodes

**Figure 4.**
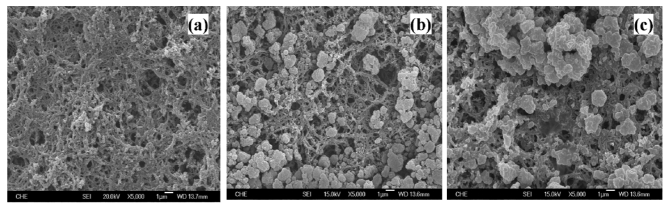
The SEM images of the surfaces of (a) PANI/Pt, (b) SBA-15/PANI/Pt, and (c) GA/SBA-15(HRP)/PANI/Pt electrodes.

After depositing the SBA-15 on the electrochemically synthesized PANI/Pt electrode, SEM was employed to illustrate the surface morphology of the constructed electrodes. As shown in Figure 4a, the SBA-15 formed aggregations with the size of several micrometers, in which stably attached to the surface or filled in the inner matrix of fibrous PANI film. Figure 4(c) presents the surface morphology of the constructed GA/SBA-15(HRP)/PANI/Pt electrode, where SBA-15 particles appeared to be covered by the thin layer formed by glutaraldehyde. If glutaraldehyde was not applied (data not shown), the electrode exhibited the similar surface morphology as that of SBA-15/PANI/Pt electrode in Figure 4(b), however, the stability was relatively poorer than that of GA/SBA-15(HRP)/PANI/Pt electrode and the comparison was further discussed later.

### 2.4. Electrochemical Response of Electrodes towards H2O2

**Figure 5.**
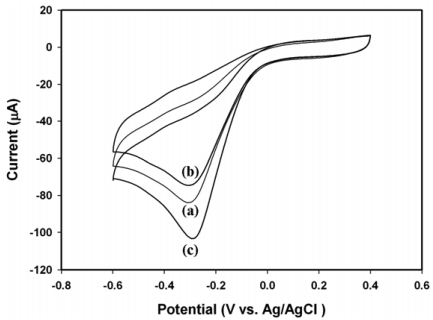
The cyclic voltammograms of: (a) PANI/Pt, (b) GA/SBA-15/PANI/Pt, and (c) GA/SBA-15(HRP)/PANI/Pt in response to 18.5 mM of H2O2 in 0.1 M phosphate buffer (pH 6.2) with a scan rate of 20 mV/s.

Figure 5 shows the cyclic voltammograms of electrodes in response to 18.5 mM of H_2_O_2_ in 0.1 M phosphate buffer (pH 6.2). The PANI modified Pt electrode displayed considerable cathodic response to H_2_O_2_, as represented by line (a) in Figure 5, and was similar to that reported in our previous publication [1]. The deposition of unloaded SBA-15 on the PANI film decreased the peak current [line (b) in Figure 5], implicating the reduction of conductivity due to the electronically insulating SBA-15 particles on the PANI film. However, the immobilization of SBA-15(HRP) on the PANI film dramatically enhanced the cathodic response as expected [line (c) in Figure 5]. According to the results shown in Figures 1 and 2 as well as Table 1, the mesopores of SBA-15 provided the electro-active proteins with a unique environment that might prevent protein aggregation during immobilization. A recent publication has demonstrated that the SBA-15 mesoporous materials accelerated the electron transfer between the entrapped enzyme and electrode [29]. The similar conclusions were also made for other mesoporous silica [30-32] and the electron hopping mechanism was proposed to facilitate the electron transfer inside silica mesopores [29,33-35].

**Figure 6.**
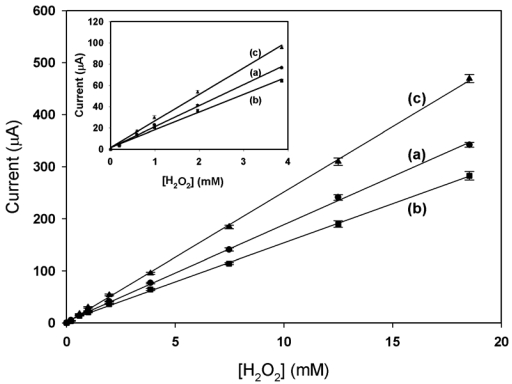
The linear calibrations of (a) PANI/Pt, (b) GA/SBA-15/PANI/Pt, and (c) GA/SBA-15(HRP)/PANI/Pt with various concentration of H2O2 in 0.1 M phosphate buffer (pH 6.2). The data were collected from three separate experiments on three individual sensors.

Meanwhile, the novel constructed electrode exhibited a nice linear correlation with H_2_O_2_ in the range of 0.02 to 18.5 mM [*R*^2^ = 0.997, line (c) in Figure 6]. The sensitivity of GA/SBA-15(HRP)/PANI/Pt electrode towards H_2_O_2_ was obtained as 89.46 μA·mM^-1^·cm^-2^, which was better than those of 66.21 μA·mM^-1^·cm^-2^ for PANI/Pt and 53.71 μA·mM^-1^·cm^-2^ for SBA-15/PANI/Pt electrodes, as shown by lines (a) and (b) in Figure 6, respectively. Although the amount of HRP actually entrapped in the GA/SBA-15(HRP)/PANI/Pt electrode was much less than that employed for direct immobilization of HRP on the PANI film (GA/HRP/PANI/Pt electrode) as we previously reported [1], their cathodic response and sensitivity were all comparable. The inset in Figure 6, also displayed their linear correlations with H_2_O_2_ in the range of 0.02 to 4 mM, and the limit detection of GA/SBA-15(HRP)/PANI/Pt sensor was about 10 μM.

### 2.5. The Stability of Constructed Electrode

**Figure 7.**
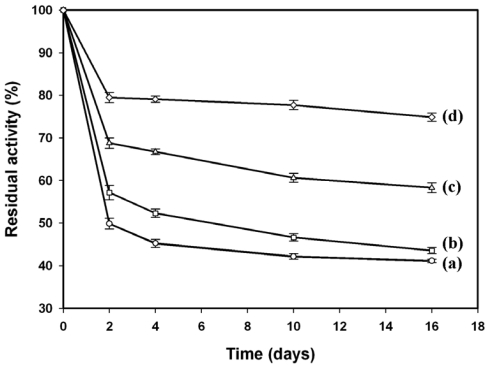
The current responses of (a) PANI/Pt, (b) GA/HRP/PANI/Pt, (c) SBA-15(HRP)/PANI/Pt, and (d) GA/SBA-15(HRP)/PANI/Pt electrodes to 1.96 mM H2O2 in a 16-day period with multiple measurements. The data were collected from three separate experiments on the same sensor.

It has been reported that the electrochemical synthesized PANI film on the Pt electrode may be unstable during the sequential cyclic voltammetric measurements, in which can be improved by the implanted bovine serum albumin [1]. The same results are shown in Figure 7, where the cathodic current towards 1.96 mM H_2_O_2_ plunged down near 50% for PANI/Pt (line a) and 43% for GA/HRP/PANII/Pt (line b), respectively, with the second measurement. By the fifth measurement of a 16-day period, both electrodes lost near 50% of their initial responses. However, the cathodic current dropped about 30% for SBA-15(HRP)/PANI/Pt (line c) and 20% for GA/SBA-15(HRP)/PANI/Pt (line d) with the second measurement, but the decay observed for GA/SBA-15(HRP)/PANI/Pt during the subsequent measurements was insignificant, therefore we concluded that glutaraldehyde might be able to stabilize the entrapped HRP through crosslinking or forming the thin film, as indicated by Figure 4c. Meanwhile, the improvement of PANI film stability was probably ascribed to the filling of SBA-15 in the matrix of PANI network as shown in Figures 4(b) and (c).

The entrapment of enzyme in the mesopores of SBA-15 has been proposed to occur by means of electrostatic interaction, simple adsorption, and entrapment [36], therefore the interactions between enzyme and the inorganic inner pore surface of SBA-15 were not tight enough. In this study, the loaded SBA-15 was rinsed with phosphate buffer for several times until no measurable leaching of HRP was detectable, based on the Bradford assay as well as ABTS enzymatic assay. However, significant reductions of the cathodic responses for both SBA-15(HRP)/PANI/Pt and GA/SBA-15(HRP)/PANI/Pt were still observed after the initial cyclic voltammetric measurement, in which were partially resulted from the exclusion of enzyme molecules by the electrostatic repulsion. Nevertheless, the SBA-15 loaded with HRP provided the PANI/Pt electrode with not only an enhanced sensitivity but also an improved stability, in which could be further improved by employing glutaraldehyde.

## 3. Experimental Section

### 3.1. Chemicals

Horseradish peroxidase (HRP), copolymer poly(ethylene glycol)-block-poly(propylene glycol)- block-poly(ethylene glycol) (EO_20_PO_70_EO_20_, Pluronic P123 with the molecular weight of 5,800), 1,3,5-Trimethylbenzene (TMB), and tetraethyl orthosilicate (TEOS, 98%) were commercially available from Sigma-Aldrich Corp. (St. Louis, MI, USA). Glutaraldehyde (GA, 25%, v/v), hydrogen peroxide (35%, v/v), aniline monomer were obtained from Merck KGaA (Darmstadt, Germany). All other reagents used for buffer and standard solution preparation were were of analytical grade and purchased from various commercial sources.

### 3.2. Preparation of SBA-15 Mesoporous Silica

SBA-15 was prepared according to the procedure described by Lettow *et al.* [17] with minor modifications. In a routine preparation, 2 g of Pluronic P123 was completely dissolved in 75 mL of 1.6 M HCl at 35 °C with stirring, 0.2 g of TMB as a swelling agent was then added with stirring for 1 h. Later on, 4.25 g of TEOS was added to serve as the silica source. The mixture was further stirred for 24 h at 35 °C and aged without stirring for 48 h at 100 °C, while the solids were recovered by filtration and air-dried at room temperature. Finally, the product was calcined under 600 °C for 2 h to remove remaining triblock copolymer.

### 3.3. Characterizations of SBA-15 Mesoporous Silica

The X-ray diffraction (XRD) measurements of calcined SBA-15 were performed on a X'Pert MPD pro diffractometer (PANalytical, ALMELO, The Netherlands) using CuK_α_ radiation (λ = 1.5418 Å) in the range of 0.3 ∼ 3° 2θ with step of 0.03° per second. The specific surface area, total pore volume, and pore size distribution of the SBA-15 were estimated by nitrogen adsorption and desorption under 77K with a Micromeritics ASAP 2020 instrument (Norcross, GA, USA), and BJH pore diameter was obtained based on the Barrett-Joyner-Halenda (BJH) calculation. The mesoporous structure was imaged by a JEOL transmission electron microscope (TEM, JEM-2010 at 200 kv, Tokyo, Japan) and by a JEOL field emission scanning electron microscope (SEM, JSM-7000F at 15 kV, Tokyo, Japan).

### 3.4. Assay of Protein Activity

The HRP activity assay was performed by the 1-Step™ ABTS protocol (PIERCE Chemical Co., Rockford, IL, USA) according to manufacturer's procedure. In brief, one milliliter of assay mixture contained 150 μL of 1-Step™ ABTS reagent and 1,850 μL of diluted HRP solution in 0.1 M phosphate buffer (pH 6.2). The mixture was incubated at room temperature for 15 minutes and the reaction was stopped by adding 100 μL of stop solution containing 1% SDS. The absorbance was measured at 410 nm with a Genesys 2 UV-vis spectrophotometer (Rochester, NY, USA). For HRP immobilized in SBA-15, the reaction was carried out in a 1.5 mL Eppendorf tube and the final product was centrifuged with 7,000 rpm for 5 minutes. The supernatant was subjected to the measurement of absorbance at 410 nm.

### 3.5. Immobilization of HRP

Prior to immobilization, enzyme stock solution was prepared by dissolving 16.8 mg of HRP (5,000 units) in 1 mL of 0.1 M phosphate buffer (pH 6.2), and then was aliquoted and stored at -80 °C. To perform enzyme immobilization, 20 μL of HRP stock solution (100 units) was first diluted to 200 μL by 0.1 M phosphate buffer (pH 6.2) and subsequently 10 mg of SBA-15 was suspended in the enzyme solution for 1 h at 4 °C on a rotator. The loaded SBA-15 (SBA-15(HRP)) was recovered by centrifugation with 7,000 rpm for 5 min and the supernatant was also collected for protein analysis. The SBA-15(HRP) was washed by 0.1 M phosphate buffer for at least 5 times, and was finally resuspended in 10 mL of 0.1 M phosphate buffer (pH 6.2). The amount of HRP immobilized on SBA-15 was determined according to Bradford assay with a Genesys 2 UV-vis spectrophotometer (Rochester, NY, USA) and HRP activity was assessed by 1-Step™ ABTS method (PIERCE Chemical Co., Rockford, IL, USA) according to the manufacturer's procedure.

### 3.6. Fabrication of the Biosensor

The PANI/Pt electrode was prepared following a procedure similar to that described our previous publication [1]. The Pt/ceramic electrode with a desired pattern (area: 0.28 cm^2^) was constructed by sputtering platinum to a ceramic plate (area: 2 cm^2^) with a shadow mask for 600 sec on a sputter instrument (JFC-1200, JEOL, Tokyo, Japan), then washed with 3M NaOH and 3M HCl, rinsed with water, and finally dried under 50 °C for one hour. A certain amount of aniline was then electropolymerized onto the Pt/ceramic base by immersing the working electrode into a solution containing 1 M HCl, 0.1 M aniline, whilst the potential was swept from 0.0 to 1.0 V for certain cycles under an ambient condition. The fashioned PANI/Pt electrodes were then immersed in 0.1 M phosphate buffer (pH 4.0) and reduced at -0.5 V for 20 min to remove the remaining chloride ions that were possibly embedded in the polymer matrix. It was then oxidized in the same phosphate buffer at 0.6 V for 10 min. To construct SBA-15/PANI/Pt and SBA-15(HRP)/PANI/Pt electrodes, 20 μL SBA-15 or SBA-15(HRP) solution was carefully dropped onto the surface of PANI/Pt electrode and air-dried at room temperature. The electrodes were then stabilized by dropping a 2.5% (v/v) glutaraldehyde solution, and incubated at 4 °C for overnight to form the covalent linkages. Afterward, the constructed electrode was rinsed with PBS buffer (pH 5.6) thoroughly and stored in a 4 °C refrigerator. The measurements were preferred to be performed within 48 hours.

### 3.7. Electrochemical Measurement

A PC-controlled CHI621B electrochemical analyzer (CH Instruments, Austin, TX, USA) was employed to run cyclic voltammetric experiments for electrode preparation and hydrogen peroxide measurement. All experiments were performed in a miniature electrochemical cell using a modified PANI/Pt electrode (area: 0.28 cm^2^) as the working electrode, a platinum wire as the auxiliary electrode, and an Ag/AgCl (3M NaCl) electrode as the reference electrode. The reductions of H_2_O_2_ on electrodes were quantified with cyclic voltammetry in 0.1 M phosphate buffer (pH 6.2). The buffer had undergone deoxygenation with highly pure nitrogen for 20 min before a certain amount of H_2_O_2_ was added. During the calibration, pure nitrogen gas was gentle purged on the surface of the sample solution to create an anaerobic atmosphere.

## 4. Conclusions

We have presented a new strategy for the fabrication of hydrogen peroxide biosensor based on entrapping HRP in mesoporous SBA-15 and depositing on a PANI modified Pt electrode. Our results further indicated that the synthetic SBA-15 particle possessed well-defined pore geometry and high internal surface area that was able to enhance the physical adsorption of enzyme molecules. The proper pore size distribution of SBA-15 was suitable for the entrapment of HRP and maintaining its bioactivity. Meanwhile, the novel GA/SBA-15(HRP)/PANI/Pt biosensor exhibited enhanced sensitivity and a fine linear correlation between the cathodic response and the concentration of H_2_O_2_ in the range of 0.02 to 18.5 mM (*R*^2^ = 0.997). In particular, the current approach by utilizing SBA-15 to entrap HRP provided the biosensor with improved stability for multiple measurements. As shown in Figure 5, the lost of current response was mainly occurred during the initial cyclic voltammetric measurement, suggesting a few of HRP molecules were excluded by the applied potential.

Furthermore, the pore size of SBA-15 can be easily adjusted by controlling the synthesis conditions in the presence of pore expanding reagents, such as 1,3,5-trimethylbenzene (TMB) [21,37-39], and biomolecules with different molecular mass can be entrapped in SBA-15 of proper pore size accordingly. Meanwhile, the internal surface of SBA-15 has been successfully modified by various organic functional groups, such as amine, thiol, and carboxylic acid, thereby providing additional improvement to minimize the leaching of biomolecules [20]. Nevertheless, the entrapment of biomolecules in SAB-15 may provide new aspects of enhancing the performance of enzyme-based biosensors, in particular offering better stability and multiple usages.

## Figures and Tables

**Table 1.** Pore characterizations of SBA-15 and SBA-15(HRP).

**Sample**	*A***_BET_ (m^2^/g)**	*V***_total_ (cm^3^/g)**	*a***_0_ (Å)**	*D***(Å)**
SBA-15	708.5	0.92	12.56	76
SBA-15(HRP)	632.3	0.85	12.16	76

***A*_BET_**: total specific surface area; ***V*_total_**: total pore volume; ***a*_0_**: lattice parameter; **D**: BJH pore diameter.
